# Tropical forest soil carbon stocks do not increase despite 15 years of doubled litter inputs

**DOI:** 10.1038/s41598-019-54487-2

**Published:** 2019-12-02

**Authors:** Emma J. Sayer, Luis Lopez-Sangil, John A. Crawford, Laëtitia M. Bréchet, Ali J. Birkett, Catherine Baxendale, Biancolini Castro, Chadtip Rodtassana, Mark H. Garnett, Lena Weiss, Michael W. I. Schmidt

**Affiliations:** 1 Lancaster Environment Centre, Lancaster University, Lancaster, LA1 4YQ UK; 2 Smithsonian Tropical Research Institute, P.O. Box 0843-03092, Balboa, Ancon, Panama Republic of Panama; 3 Department of Botany, Faculty of Science, Chulalongkorn University, Bangkok, Thailand; 4 NERC Radiocarbon Facility (East Kilbride), Scottish Enterprise Technology Park, East Kilbride, Glasgow, G75 0QF UK; 5 Department of Geography, University of Zürich, Winterthurerstr. 190, 8057, Zürich, Switzerland; 6 Present Address: Teagasc, Environmental Research Centre, Johnstown Castle, Co., Wexford, Y35 TC97 Ireland; 7 Present Address: Department of Biology, University of Antwerp, B-2610 Wilrijk, Belgium

**Keywords:** Climate-change ecology, Carbon cycle, Carbon cycle, Tropical ecology

## Abstract

Soil organic carbon (SOC) dynamics represent a persisting uncertainty in our understanding of the global carbon cycle. SOC storage is strongly linked to plant inputs via the formation of soil organic matter, but soil geochemistry also plays a critical role. In tropical soils with rapid SOC turnover, the association of organic matter with soil minerals is particularly important for stabilising SOC but projected increases in tropical forest productivity could trigger feedbacks that stimulate the release of stored SOC. Here, we demonstrate limited additional SOC storage after 13–15 years of experimentally doubled aboveground litter inputs in a lowland tropical forest. We combined biological, physical, and chemical methods to characterise SOC along a gradient of bioavailability. After 13 years of monthly litter addition treatments, most of the additional SOC was readily bioavailable and we observed no increase in mineral-associated SOC. Importantly, SOC with weak association to soil minerals declined in response to long-term litter addition, suggesting that increased plant inputs could modify the formation of organo-mineral complexes in tropical soils. Hence, we demonstrate the limited capacity of tropical soils to sequester additional C inputs and provide insights into potential underlying mechanisms.

## Introduction

Soil organic carbon (SOC) storage depends primarily on the balance between inputs of plant material and losses of SOC through decomposition^1^. Although current global land-surface models forecast enhanced SOC storage with increasing net primary productivity (NPP), there is mounting evidence that nutrient limitation and feedbacks between above- and belowground processes will undermine this straightforward relationship^2–4^. Uncertainties in projected future SOC storage are particularly high because our mechanistic knowledge of soil C sequestration is deficient^5^. Whereas most Earth System Models consider NPP, soil temperature and moisture as the main drivers of SOC storage^6^, the stabilisation of SOC is governed by biological, physical, and chemical processes^5,7,8^, including microbial activity^9^, physical protection within soil aggregates^10^, and chemical protection via associations with soil minerals^11^. Indeed, soil geochemistry may be more important for SOC storage than climate, particularly in warm, humid regions with rapid C turnover^12^, such as tropical forests. However, as highlighted in the recent IPCC Special Report on Climate Change and Land, the sensitivity of mineral-associated SOC to altered carbon inputs is highly uncertain, which constrains projections of future land-climate feedbacks^13^.

Tropical forests play a greater role in the global C cycle than any other terrestrial ecosystem, and yet we know very little about SOC dynamics and storage in tropical forests, which creates large uncertainties in model projections of future C sequestration^14^. Projected increases in plant productivity in tropical forests could sequester vast amounts of C in aboveground biomass, but additional plant inputs to the soil could also affect SOC storage by stimulating the microbial decomposition and turnover of older, stored SOC through so-called priming effects^15,16^. Experiments in temperate and tropical forests have demonstrated sustained release of SOC by priming after several years of increased plant inputs^16–18^, implying that enhanced NPP will not necessarily entail a corresponding net gain in SOC storage^2,16,19^. The role of feedbacks such as priming effects in regulating belowground C sequestration merits further attention because a much larger proportion of SOC may be susceptible to microbial attack than previously assumed^20^. Assessing feedbacks that influence SOC storage in highly diverse tropical forests is challenging, because soil organic matter formation is linked to litter quality as well as soil properties^2,9^ and most of our current knowledge stems from studies in temperate systems, with lower plant diversity and NPP, slower C turnover rates, and distinct soil chemistry. Given the recent focus on the C sequestration potential of soils^21^ and considering the importance of tropical forests in mitigating the global rise in atmospheric CO_2_^22^, it is essential that we establish whether changes in plant inputs have the potential to modify the processes underlying the formation and storage of SOC in tropical forests.

Here, we advance current understanding of SOC storage under global change by quantifying the impact of increased litter inputs in old-growth lowland tropical forest in Panama, Central America. We assessed SOC storage and bioavailability after 13–15 years of monthly litter addition (L+) treatments to large-scale experimental plots^23^. We characterised SOC fractions along a gradient of potential availability to microbial decomposers and, as soil organic matter represents a continuum of progressively decomposing compounds^8^, we refer to these fractions as discrete pools of C only to aid interpretation. We further define stabilisation as the reduction in potential loss of SOC^11,24^, and therefore consider decreasing bioavailability or solubility as an indicator of SOC stability. We also considered the role of organo-mineral associations in determining the size and turnover of SOC pools in the tropics^12,25^ by separating ‘accessible SOC’, which may have some degree of protection by soil aggregates but is considered largely available to microbes^11^, from mineral-associated SOC, which is protected from microbial attack to varying degrees by associations with soil minerals. We further divide the mineral-associated SOC fraction into ‘intermediate SOC’, which is weakly stabilised by associations to mineral surfaces, and ‘resistant SOC’ that is more strongly sorbed to soil minerals^26^. Finally, we consider ‘highly resistant SOC’ as largely unavailable to microbial decomposers because it is bound within large, stable organo-mineral complexes, which are generally insoluble^25^.

We used four distinct but complementary approaches to test two alternative hypotheses, based on previous work at the study site that demonstrated substantial release of SOC as CO_2_ in response to the litter addition treatments^16^:

H1) Most of the extra organic C inputs with litter addition have been gradually incorporated into the soil, resulting in enhanced storage of mineral-associated SOC.

H2) Extra inputs of new litter-derived C and priming of older SOC have resulted in greater overall bioavailability of SOC and limited additional storage in mineral-associated fractions.

**Figure 1:**
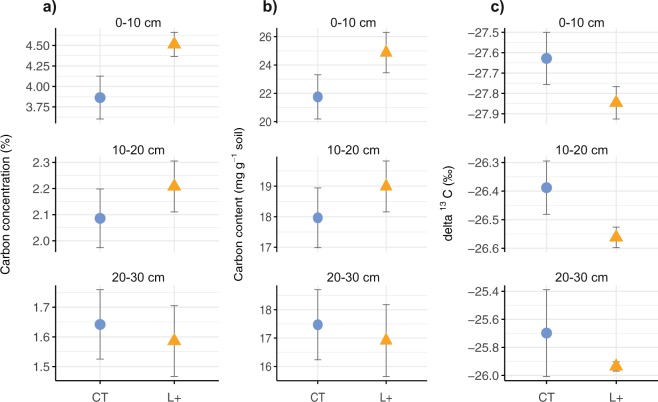
Changes in soil organic carbon (**a**) concentrations, (**b**) content, and (**c**) δ^13^C values in 10-cm increments from 0–30 cm depth after 15 years of litter addition treatments in a lowland tropical forest soil in Panama, Central America, where L+ is litter addition (triangles) and CT is controls (circles); means and standard errors are shown for *n* = 4 per treatment; soil mass was corrected for differences in bulk density.

First, we quantified changes in total SOC content and the δ^13^C of SOC at 0–30-cm depth after 15 years of continuous monthly litter addition treatments (see Methods). An overall increase in SOC concentrations in the L+ plots (*χ*^2^ = 57.6, *p* < 0.001) was largely due to *c*. 17% higher SOC concentrations at 0–10 cm, as there were no differences between treatments at 10–20 cm or 20–30 cm (Fig. 1a). After correction for variation in bulk density, we found no significant increase in SOC content in the L+ plots at any depth increment (Fig. 1b). As expected, the δ^13^C of SOC increased with soil depth, but the values were more negative in the L+ plots to 20-cm depth (*χ*^2^ = 3.93, *p* = 0.048; Fig. 1c), which suggests that organic matter in the L+ plots is in a less advanced state of decomposition^27^ compared to the controls. The lower mean δ^13^C of SOC in the L+ plots can be at least partly explained by increased incorporation of fresh organic C^28^, but it also provides a first indication of greater overall bioavailability of SOC with litter addition.

**Figure 2:**
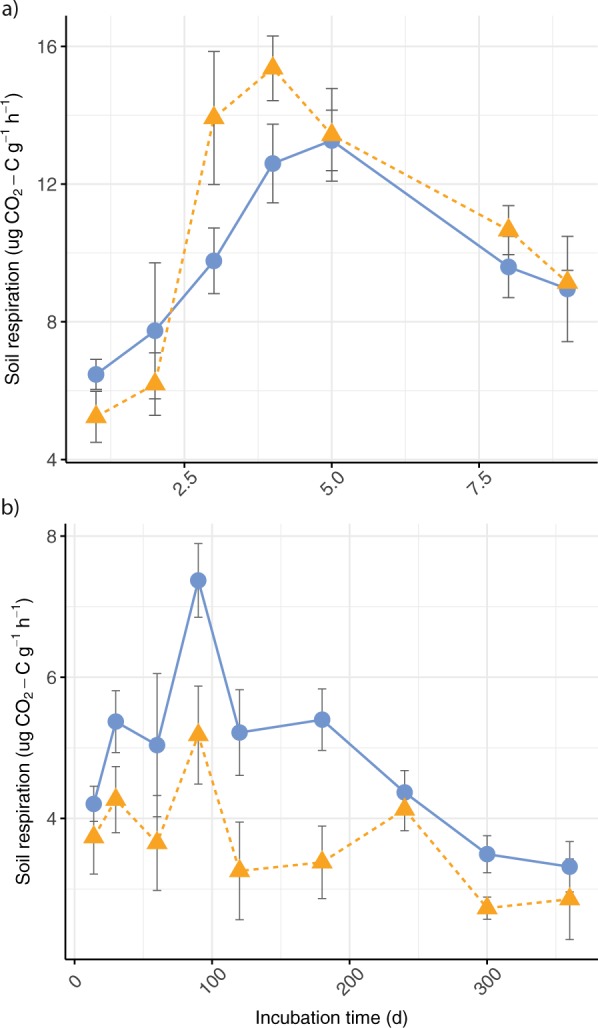
Soil respiration during (**a**) the first 10 days and (**b**) from 10–360 days of a long-term incubation of mineral soil (0–10 cm depth), showing CO_2_ efflux from soils collected after 13 years of litter addition (L+; dashed line) and control treatments (CT; solid line) in lowland tropical forest in Panama, Central America; incubation means and standard errors are shown for *n* = 4 per treatment and time-point; note that the y-axis scales differ between panels.

We then tested our hypothesis of increased bioavailability of SOC in L+ plots directly by incubating soil samples from the experimental plots under controlled conditions for 360 days without C inputs and measuring CO_2_ efflux to assess the microbial availability of SOC (Methods). This approach assumes that accessible SOC will be mineralised and respired rapidly, and intermediate SOC will be utilised once the most available substrate has been depleted, leaving resistant or highly resistant SOC that is unavailable to microbes^29^. Litter addition altered the pattern of soil respiration over time (*χ*^2^ = 29.7, *p* < 0.001): during the first 10 days, CO_2_ efflux was *c*. 13% higher in L+ soils compared to controls (Fig. 2a), reflecting the higher proportion of accessible SOC. However, CO_2_ efflux from L+ soils declined after the first two weeks and was significantly lower than the controls for the remainder of the year (*χ*^2^ = 24.0, *p* < 0.001; Fig. 2b), which suggests that the amount of intermediate SOC has declined relative to controls. The amount of SOC remaining in the L+ soils at the end of the experiment (6.9 ± 0.2 mg g^−1^) was remarkably similar to the controls (6.8 ± 0.4 mg g^−1^), indicating that resistant or highly resistant SOC was unaffected by litter addition, and that greater total SOC loss from the L+ soils during the one-year incubation (*t* = −3.15, *p* = 0.049) can be attributed to rapid mineralisation of accessible SOC. Hence, our incubation experiment supports our hypothesis of greater overall bioavailability of SOC with litter addition.

**Figure 3:**
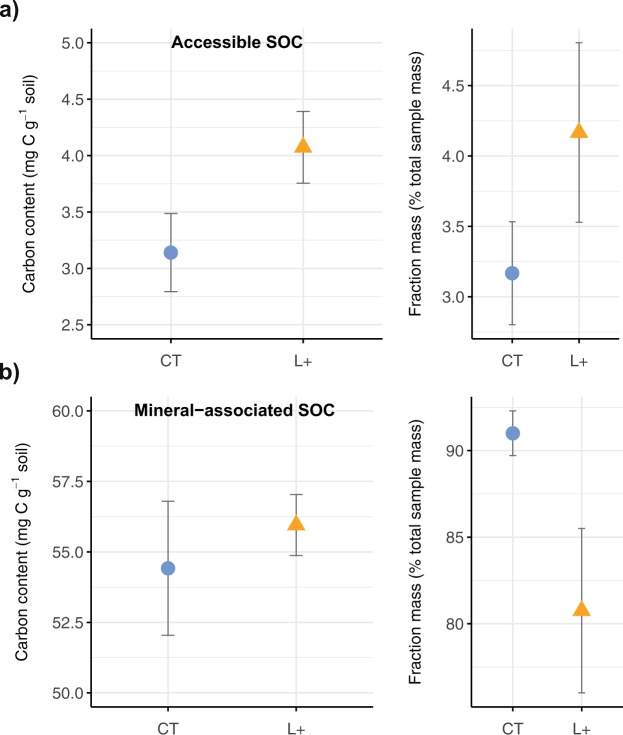
Changes in the carbon content and mass of soil organic carbon (SOC) fractions at 0–10 cm depth after 13 years of litter addition (L+; triangles) compared to control soils (CT; circles) in a lowland tropical forest soil in Panama, Central America, showing the carbon content (left-hand panels) and the proportion of each fraction relative to the total sample mass (right-hand panels) of (**a**) the accessible SOC fraction (2000–20 μm) and (**b**) the mineral-associated SOC fraction (<20 μm); means and standard errors are shown for *n* = 4 per treatment; soil mass was corrected for differences in bulk density.

We used physical fractionation to quantify pools of accessible (2000–20 µm) and mineral-associated SOC (<20 µm; Methods) in soils collected from the field experiment. The majority of SOC was associated with soil minerals, which is common in tropical soils because unprotected organic matter is decomposed rapidly^25^. Nonetheless, litter addition influenced the distribution of SOC among particle size fractions (*χ*^2^ = 106.0, *p* < 0.001), whereby the amount of accessible SOC was greater in the L+ plots (Fig. 3a) but the mineral-associated SOC fraction was remarkably similar between L+ plots (56.0 ± 1.1 mg C g^−1^) and controls (54.4 ± 2.4 mg C g^−1^; Fig. 3b). Consequently, the relative contribution of mineral-associated SOC to total SOC was lower in the L+ plots (81 ± 4.8%; Fig. 3b) than in the controls (91 ± 1.3%; *t* = −2.2, *p* = 0.066; Fig. 3a). These findings raise three possibilities: i) the incorporation of fresh litter-derived C into the soil in the L+ plots has outpaced its subsequent stabilisation by association with soil minerals, ii) the capacity of the soil to sequester additional mineral-associated SOC is saturated^11^, or iii) the release of mineral-associated SOC, e.g. by priming effects, has offset the storage of new SOM. We explored these possibilities using sequential chemical extractions^30,31^ to assess the distribution of mineral-associated SOC along a gradient of increasing sorption strength (Methods).

**Figure 4:**
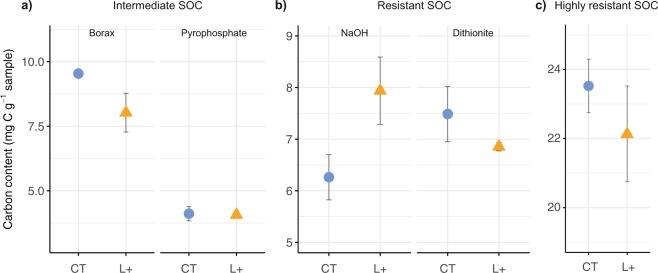
Changes in the carbon content of mineral-associated soil fractions (<20 μm) after 13 years of litter addition (L+; triangles) treatments compared to controls (CT; circles) in lowland tropical forest in Panama, Central America showing different fractions extracted by sequential chemical extractions: (**a**) intermediate soil organic carbon (SOC), represented by sodium tetraborate (borax) and sodium pyrophosphate extractions; (**b**) resistant SOC represented by sodium hydroxide (NaOH) and dithionite extractions; (**c**) highly resistant SOC remaining after chemical extraction; means and standard errors are shown for *n* = 4 per treatment; soil mass was corrected for differences in bulk density.

Litter addition altered the distribution of mineral-associated C between intermediate and resistant fractions (*χ*^2^ = 186.8, *p* < 0.001). Weakly stabilised intermediate SOC (tetraborate extraction) had declined in the L+ plots compared to the controls (*p* = 0.095), resulting in an overall decrease in intermediate SOC with litter addition even though the amount of intermediate SOC extracted by pyrophosphate was unaffected (Fig. 4a). This counterintuitive decrease in intermediate SOC with litter addition substantiates our interpretation of the lower respiration in L+ compared to control soils in our incubation experiment (Fig. 2b) and could be explained by competitive adsorption, where weakly sorbed compounds are released from mineral surfaces by sorptive fresh organic inputs via exchange reactions^24,30^. It is thus conceivable that intermediate SOC pools are the source of the CO_2_ we attributed to priming effects in the field^16^, and this possibility warrants further investigation.

Despite more than a decade of double litter inputs, the amount of resistant and highly resistant SOC was unaffected by litter addition. Sodium hydroxide (NaOH) extraction revealed a small increase in resistant SOC with litter addition (*p* = 0.077; Fig. 4b), suggesting that the soil at our study site has the capacity to sequester additional organic inputs within the time-frame of the field experiment, but this was offset by a non-significant decrease in the amount of resistant SOC associated specifically with iron oxides (dithionite extraction Fig. 4b). This is surprising because organo-mineral complexes involving iron oxides are thought to be resistant to microbial attack due to their extremely low solubility^25^, but it could also indicate changes in the direct sorption of litter-derived C to oxyhydroxides^32^. Measurements of soil nutrient availability using ion exchange resins provide corroborating evidence for this possibility, as they revealed a two-fold increase in iron cations in the L+ plots relative to the controls (Sayer *et al*., unpublished data). Finally, we found no indication that highly resistant SOC was affected by litter addition (Fig. 4c). Overall, the altered distribution of mineral-associated SOC fractions provides a potential explanation for the lack of additional SOC storage in the L+ plots, whereby additional litter inputs influence the formation or dissolution of organo-mineral associations that underpin the storage of SOC. Here, the decline in intermediate SOC (Fig. 4a) is particularly striking, as it suggests that litter addition could disrupt weaker organo-mineral bonds and indicates a possible weak link in the progressive stabilisation of SOC in these soils. Elucidating whether the observed changes in mineral-associated SOC fractions are a result of competitive adsorption or other changes in soil geochemistry is an important avenue of future investigation, which will help address current knowledge gaps about feedbacks between plant inputs and SOC storage.

Finally, radiocarbon (^14^C) analyses of mineral-associated SOC corroborated the findings of our experiments. We found that a substantial amount of mineral-associated SOC in our tropical forest soil was fixed within the timeframe of the field experiment (within the last 5–15 years based on levels of ‘bomb-^14^C’^33^). Hence, if a greater amount of litter-derived C had been stabilised in the L+ plots, we would expect ‘younger’ mineral-associated SOC compared to the controls. However, the concentrations of ^14^C in the L+ samples were remarkably similar to the controls (106.2 ± 0.33 and 106.1 ± 0.51% modern, respectively). Assuming SOC storage is the result of the progressive stabilisation of organic matter^8^, one possible interpretation of this result is that litter addition has boosted the turnover of ‘younger’ intermediate SOC but has had a limited effect on the transfer of SOC to ‘older’ resistant and highly resistant pools, resulting in a similar overall age of mineral-associated SOC.

Taken together, the results of our four approaches provide little evidence to support the hypothesis of increased SOC storage after 13–15 years of litter addition. Instead, biological, physical and chemical fractionation of SOC indicated gains in accessible SOC with litter addition but a decline in intermediate mineral-associated SOC, which is consistent with our second (alternative) hypothesis. Hence, we propose that increased incorporation of new litter-derived organic C in the L+ plots and concomitant losses of weakly stabilised (intermediate) mineral-associated SOC resulted in greater overall bioavailability of the total SOC pool.

## Implications of the Study

We found remarkably little evidence to support current projections of greater SOC storage with enhanced plant productivity over decadal timescales. Indeed, our study demonstrates for the first time that increased tropical forest litter inputs can alter the processes involved in the stabilisation of SOC via association with soil minerals, which is particularly important for SOC storage in the humid tropics^12,25^. The possibility that litter addition influences SOC stabilisation by iron oxide complexes is an intriguing avenue for future work, because strong iron oxide complexes play an important role in stabilising SOM in soils across the tropics^25,34,35^. Hence, a common pattern is emerging in forests worldwide: studies in temperate forest ecosystems have revealed surprisingly minor increases in C storage in the mineral soil with long-term litter addition^36–38^ and free-air CO_2_ enrichment^17,39^, as well as evidence for degradation of older SOC^40^. In many temperate forest studies, the smaller plots, longer treatment application intervals, and slower C turnover could conceivably explain why litter addition treatments have had little overall influence on total SOC content. Our monthly treatments to large-scale tropical forest plots reveal that changes in the stabilisation of SOC in response to increased litter inputs can occur at an ecosystem-relevant scale.

We found that high SOC concentrations at the soil surface did not translate to greater overall SOC storage, and the limited capacity for extra inputs of organic material to enhance soil C sequestration is also demonstrated in agricultural no-till systems^41^, where increased SOC concentrations in surface layers did not extend to deeper soil layers. Experiments in other ecosystems found that greater plant diversity and higher biomass production can increase SOC concentrations in the surface horizons, but total soil profile stocks do not show conclusive trends^42–44^.

Previous work at our study site reported minor changes in C density fractions, which were largely equivalent to our results for accessible and mineral-associated SOM based on particle-size fractionation^45^. The seasonal increase in mineral-associated C stocks with litter addition at 0–5 cm depth^45^, indicates only minor changes in soil C storage close to the soil-litter interface. Hence, the rapid turnover of mineral-associated SOC at our study site and the lack of changes below 10-cm depth suggest that high biological activity at the soil surface is likely to be a key determinant of SOC storage over decadal time scales. Although it is possible that the trend towards higher SOC concentrations at the surface may extend to deeper horizons over the long-term, previous depth profiles to 2-m found no discernible changes in SOC or nutrients below 20-cm depth^46^. After a decade of treatments, we also found no indication that SOC had increased below 20-cm (Fig. 1a,b) and experiments in temperate forests have found no evidence of additional SOC storage even after 50 years of increased litter inputs^38,47^.

In conclusion, we demonstrate that interactions between litter inputs and processes involved in the stabilisation of SOC are likely to play a key role in tropical forest soil C sequestration in future and we call for further work on the underlying mechanisms.

## Methods

### Experimental design, field measurements and sample collection

We characterised changes in total SOC concentrations, and SOC fractions in a long-term litter manipulation experiment in a 40-ha area of mature lowland tropical forest in Panama, Central America. The soils at the study site are classed as oxisols^48^, with kaolinite as the dominant clay mineral^49^. Soil pH is *c*. 5.5 and total SOC content is *c*. 4–5% at 0–10 cm depth^50^. The litter manipulation experiment comprises fifteen 45-m × 45-m plots where, starting in January 2003, the litter in five L- plots is removed each month and added to five L+ plots, leaving five plots as undisturbed controls (CT)^23^. As our present study focusses on the fate of litter inputs, all subsequent sampling and analyses focus on the L+ and CT plots.

To characterise SOC in L+ and CT plots we took soil samples after 13 and 15 years of treatments. We assessed changes in the vertical distribution of SOC by collecting soil cores in 10-cm increments from 0–30-cm depth at three random locations within the inner 30-m × 30-m of each plot in May 2018. The cores for each location and depth were individually wrapped in aluminium foil, sealed in plastic bags and air-dried within eight hours of collection. To assess changes in SOC fractions, we collected six soil cores per plot at 0–10-cm depth in September 2016. The cores were pooled to give one composite sample per plot and all fractionation analyses were performed on subsamples of the same soils (*n* = 4 per treatment).

### Vertical distribution of soil carbon concentrations

We assessed the vertical distribution of SOC in 10-cm increments from 0–30 cm depth for each of the three samples per plot (72 samples in total). The soils were sieved (2-mm) and subsamples of *c*. 10 g were ground using a ball mill. Total C concentrations and the ^12^C/^13^C isotope ratio (δ^13^C) were determined on two analytical replicates of each sample by combustion and isotope ratio mass spectrometry (EA-IRMS Delta V Plus, Thermo Fisher Scientific, Bremen, Germany).

### SOC characterisation

We assessed the potential bioavailability of SOC (0–10 cm depth) using three parallel approaches to overcome the particular strengths and weaknesses of current procedures to characterise soil organic matter pools^51^.

#### Long-term incubation experiment

We tested the overall bioavailability of SOC by biological fractionation during long-term incubation of soil collected from the CT and L+ plots. We incubated 50 g sieved (2-mm mesh) air-dried mineral soil from each plot in 0.5 L glass jars. Soil water content was maintained at 50% with deionised water and the jars were incubated in the dark at 16 ± 1°C for 360 days using ventilated lids to minimize CO_2_ accumulation between measurements. Soil respiration (CO_2_ efflux) was measured using an infrared gas analyser attached to a multiplexed system (LI-8100 and LI-8150, LiCor Biosciences, Lincoln, Nebraska, USA). We took daily measurements for the first five days, then at 8, 9, and 14 days, and then every 30–60 days for 12 months in total. This approach assumes that accessible SOC will be mineralised and respired rapidly, and intermediate SOC will be utilised once the most available substrate has been depleted, leaving highly resistant SOC that is unavailable to microbes^29^.

#### Particle-size fractionation

We used particle-size fractionation to separate accessible SOC (2000–20 µm), from mineral-associated SOC (fine silt and clay fraction; <20 µm). We quantified SOC associated with four particle-size fractions (2000–200, 200–50, 50–20, and <20 µm) by wet-sieving^52^ on two analytical replicates of each soil sample after testing and adjusting the procedure using a full set of soil samples from the experimental plots. Air-dried soil samples (*c*. 20 g) were agitated with distilled water (1:2 w:v) and glass beds for 60 minutes at 160 rpm to break up soil macro-aggregates. The soil slurry was passed through a 200-µm sieve, washed thoroughly and collected into an ice-cooled glass beaker for disruption of soil micro-aggregates by ultrasonication (VCX 130 W model, 55 W power and 440 J cm^−3^ energy output), following recommended modifications for ultrasonic dispersion to limit C transfer among fractions^53,54^. The resulting slurry was passed through a sequence of two sieves (50-µm and 20-µm mesh) into a receiver. All particles retained in the sieves were transferred to pre-weighed glass beakers, dried (65 °C) and weighed; the material retained in the 200-µm sieve was also passed through 2-mm mesh to remove large particles. The slurry retained in the receiver (particle size <20-µm) was transferred into a 1-L Erlenmeyer flask, brought to volume with distilled water and resuspended with 8.7 g K_2_SO_4_ for particle flocculation overnight. The supernatant was siphoned off and the residue was transferred into glass beakers, dried and weighed.

We analysed the organic C content of i) ground subsamples of the bulk soil, ii) the soil remaining after long-term incubation, and iii) the four size fractions by combustion oxidation (Vario EL III, Elementar Analyser Systems, Hanau, Germany). We analysed three analytical replicates of bulk soil and incubated soil, and two analytical replicates of each size fraction per plot. The recovery of organic C in the particle size fractions (excluding the ‘gravel fraction’ >2000 µm) was 89 ± 1% and there were no significant differences in the proportion of C recovered among litter manipulation treatments.

The SOC within the first three particle-size fractions (2000–20 µm) corresponds broadly to particulate organic matter, which is recently incorporated material that is either unprotected or is physically protected from microbial attack by incorporation into soil aggregates^55^. The C in the <20-µm size fraction comprises mineral-associated SOC that is sorbed to mineral surfaces to varying degrees, reducing its availability to microbial decomposers^52,56^.

#### Chemical fractionation

Our second approach investigated the stabilisation of mineral-associated SOC using sequential chemical extractions at increasing pH, which enhances the solubility of SOC by promoting the desorption of organic matter from mineral surfaces^30,31^. Here, we use extraction strength to represent the energy requirement by microorganisms to access mineral-associated SOC. The first two steps involve extraction with 0.1 M sodium tetraborate at pH 9.7 and 0.1 M sodium pyrophosphate at pH 10.2; these extractions target ‘intermediate SOC’, which is weakly associated with soil minerals. Subsequently, extraction with 0.1 M sodium hydroxide (NaOH) at pH *c*. 12 solubilizes ‘resistant SOC’ that is strongly bound to active mineral surfaces and is not solubilized by the previous extractions. Finally, a second NaOH extraction after pre-treatment with 0.1 M sodium dithionite reduces the iron in oxides, hydroxides and sesquioxides, thus solubilizing the organic molecules adsorbed to them^30,31^.

For each set of extractions, *c*. 15 g of air-dried material from the <20-µm fraction were transferred to 250-ml bottles, agitated at 160 rpm for 16 h with 80 ml extraction solution and centrifuged for 30 min at 5000 RCF. The supernatant was then decanted and the residue was extracted two more times with a 1-h agitation step. For the final extraction, the samples were agitated with 35 ml 0.1 M sodium dithionite at 40°C (pH adjusted to 8.0), centrifuged, decanted, agitated for one hour with 80 ml distilled water and centrifuged again, before extraction with 0.1 M NaOH. The supernatants from each extraction were analysed for total organic C using a TOC-L combustion analyser coupled with a TNM-L unit (Shimadzu Corp., Kyoto, Japan), except for the dithionite extractions, which were analysed in a combustion oxidizer (Vario EL III, Elementar Analyser Systems, Hanau, Germany) using 600-µl aliquots air-dried on a calcium-sulphate absorbent matrix.

The C content of all fractions was corrected for differences in bulk density and sample mass among plots. Soil bulk density at 0–10 cm depth was estimated from five soil cores per plot collected each month during the rainy season from June to October 2013; an additional five cores were collected at 0–5 cm depth to correct for any soil compaction during sampling. Consistent with previous measurements at the study site, bulk density did not differ between CT (0.56 ± 0.01 g cm^−2^) and L+ (0.55 ± 0.03 g cm^−2^) plots. Bulk density from 10–30 cm was estimated from measurements taken in soil pits at the study site^46^.

#### Radiocarbon analyses of mineral-associated soil organic carbon

To assess the residence times of mineral-associated SOC, we performed particle-size fractionation on a second set of subsamples from three plots per treatment and analysed the resulting <20-µm fractions for ^13^C and ^14^C at the NERC Radiocarbon Facility (East Kilbride, UK). Samples were combusted in sealed quartz tubes and the CO_2_ was cryogenically recovered. Stable C (^13^C) concentration was determined using isotope ratio mass spectrometry (IRMS) and results expressed relative to the Vienna PDB standard. Radiocarbon concentration was measured, following conversion of sample CO_2_ to graphite, using accelerator mass spectrometry at the Scottish Universities Environmental Research Centre (East Kilbride, UK^57^). Following convention, ^14^C results were normalised to a δ^13^C of −25‰ using the IRMS values, and expressed as %modern^57,58^. All ^14^C measurements indicated the presence of ‘bomb-^14^C’, and age was therefore interpreted by reference to records of atmospheric ^14^CO_2_ concentration^33^.

**Data analyses** were performed in R version 3.5.1^59^ using plot means for all data (*n* = 4 replicates per treatment). SOC at 0–30 cm depth (CT and L+ plots only) were assessed using linear mixed effects models with treatment and depth as fixed effects and depth nested within block as a random effect. We assessed treatment effects on *in situ* soil CO_2_ efflux and in the long-term incubations using linear mixed effects models (*lmer* function in the lme4 package^60^), with treatment as a fixed effect and time as a random effect. We compared the remaining SOC content at the end of the long-term incubations using t-tests.

We assessed changes in the relative proportions of accessible vs. mineral-associated SOC using a Generalised Linear Model (GLM) with a quasi-binomial error distribution. We then investigated whether litter treatments affected SOC associated with different physical and chemical fractions using nested linear mixed effects models with treatment, fraction, and their interaction as fixed effects and block as a random effect. Data were transformed to meet modelling assumptions where necessary. Statistics for all linear mixed effects models are given for the comparison of the final model to the corresponding null model using likelihood ratio tests and p-values for individual factors were derived by Satterthwaite’s approximation using the package *lmerTest*^61^. Due to the low number of replicates and the potential relevance of small changes in SOC, we report treatment contrasts for an overall treatment effect where *p* < 0.1.

## Data Availability

The data used in this study will be deposited on figshare upon acceptance.
