# Author Correction: Tropical forest soil carbon stocks do not increase despite 15 years of doubled litter inputs

**DOI:** 10.1038/s41598-020-69595-7

**Published:** 2020-07-23

**Authors:** Emma J. Sayer, Luis Lopez-Sangil, John A. Crawford, Laëtitia M. Bréchet, Ali J. Birkett, Catherine Baxendale, Biancolini Castro, Chadtip Rodtassana, Mark H. Garnett, Lena Weiss, Michael W. I. Schmidt

**Affiliations:** 1 Lancaster Environment Centre, Lancaster University, Lancaster, LA1 4YQ UK; 2 Smithsonian Tropical Research Institute, Balboa, Ancon, P.O. Box 0843-03092, Panama, Republic of Panama; 3 Department of Botany, Faculty of Science, Chulalongkorn University, Bangkok, Thailand; 4 NERC Radiocarbon Facility (East Kilbride), Scottish Enterprise Technology Park, East Kilbride, Glasgow, G75 0QF UK; 5 Department of Geography, University of Zürich, Winterthurerstr. 190, 8057 Zürich, Switzerland; 6 Present Address: Teagasc, Environmental Research Centre, Johnstown Castle, Co., Wexford, Y35 TC97 Ireland; 7 Present Address: Department of Biology, University of Antwerp, B-2610 Wilrijk, Belgium

Correction to: *Scientific Reports* https://doi.org/10.1038/s41598-019-54487-2, published online 02 December 2019

This Article contains errors in Figure 1 and Figure 3 due to errors within the R script used to generate these figures.

In Figure 1b, total organic carbon was not corrected for differences in bulk density, which led to incorrect values for carbon content (mg g^-1^). The values were corrected to equal sample mass. The correct Figure 1 appears below as Figure 1.

**Figure 1:**
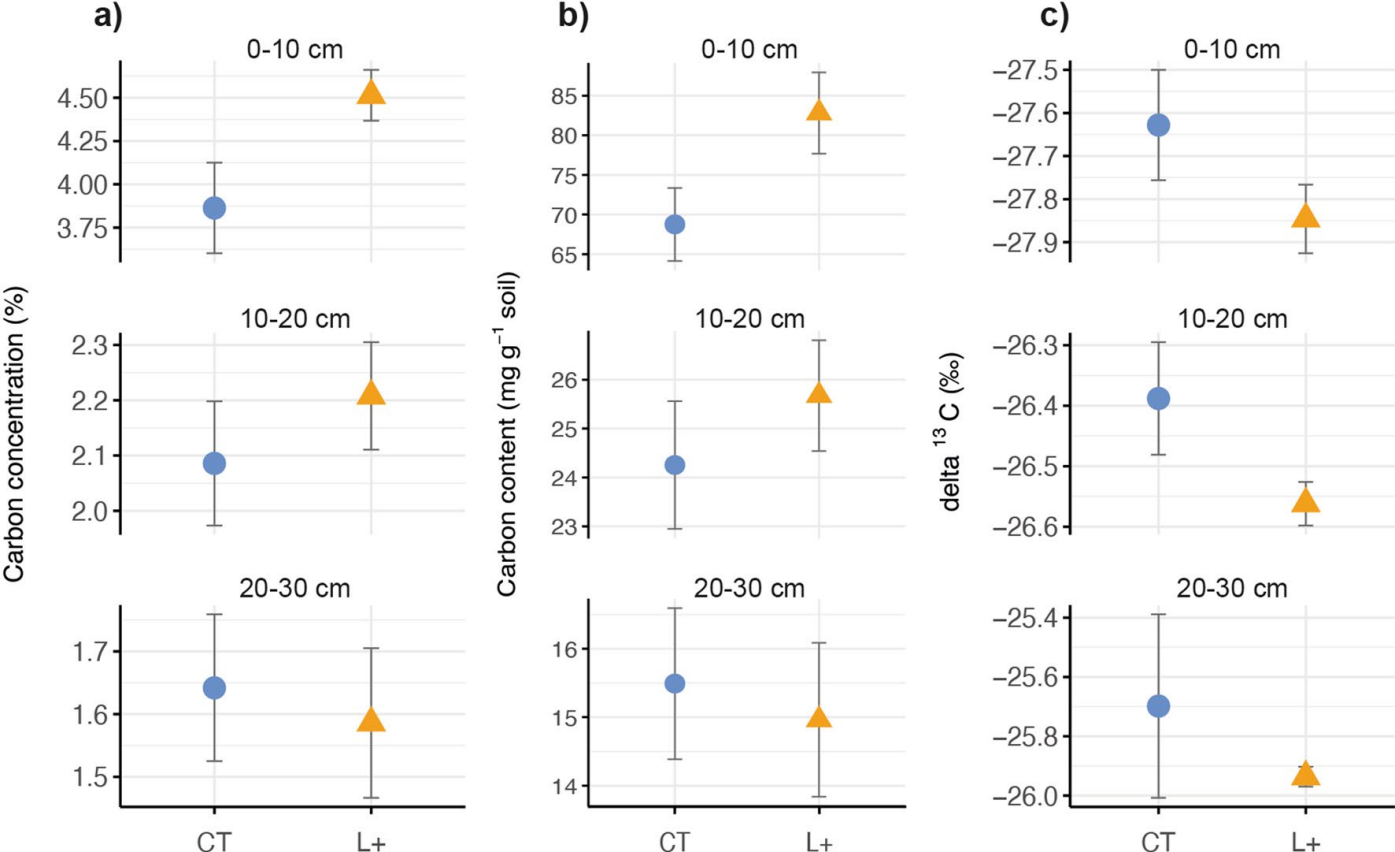
Changes in soil organic carbon (**a**) concentrations, (**b**) content, and (**c**) δ^13^C values in 10-cm increments from 0–30 cm depth after 15 years of litter addition treatments in a lowland tropical forest soil in Panama, Central America, where L+ is litter addition (triangles) and CT is controls (circles); means and standard errors are shown for *n* = 4 per treatment; soil mass was corrected for differences in bulk density.

In addition, in Figure 3a, the three individual fractions making up the total Accessible SOM were not summed before the means were calculated, which led to incorrect values for the Accessible SOC fraction. The means were recalculated and the correct Figure 3 appears below as Figure 2.

**Figure 2:**
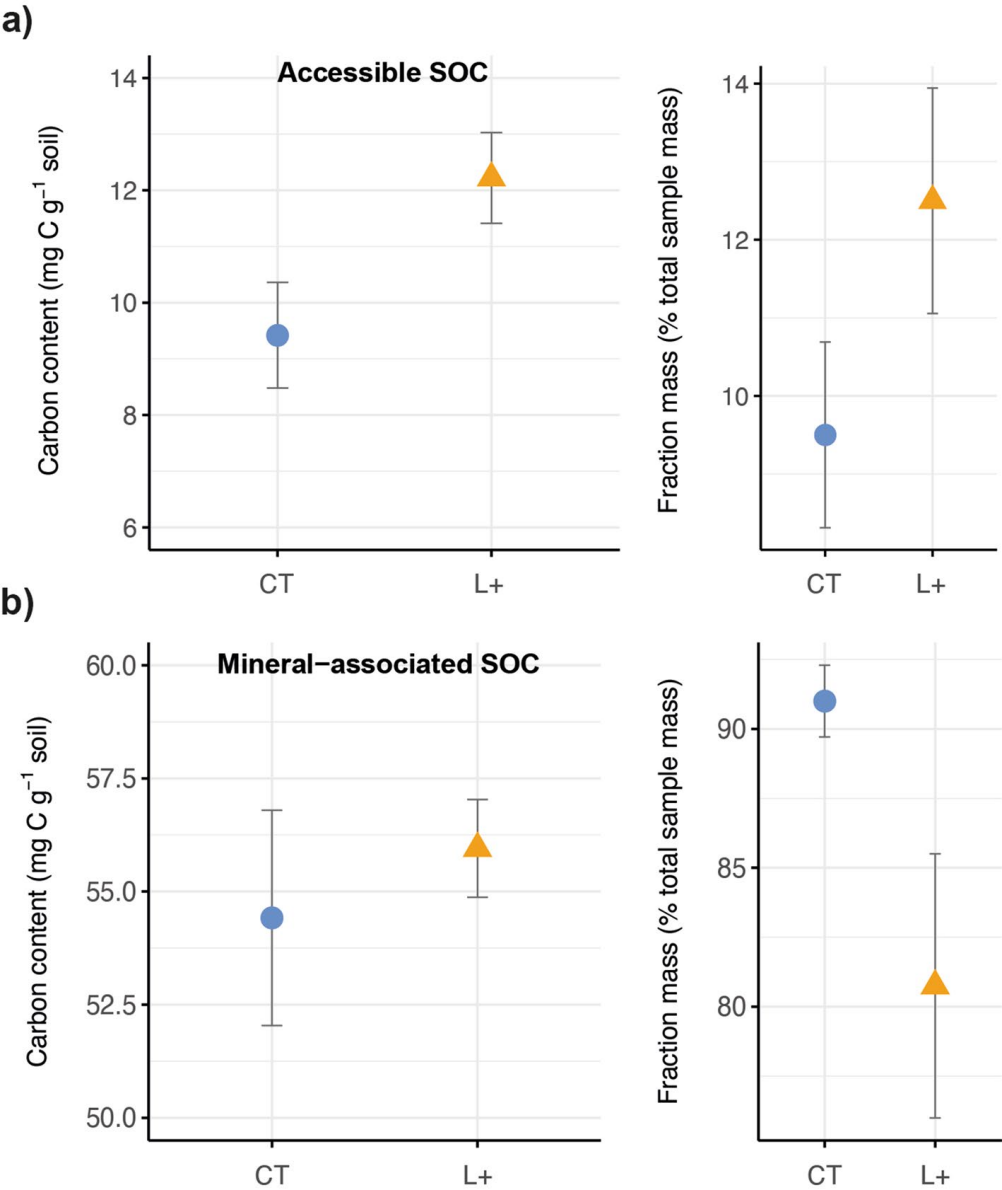
Changes in the carbon content and mass of soil organic carbon (SOC) fractions at 0–10 cm depth after 13 years of litter addition (L+ ; triangles) compared to control soils (CT; circles) in a lowland tropical forest soil in Panama, Central America, showing the carbon content (left-hand panels) and the proportion of each fraction relative to the total sample mass (right-hand panels) of (**a**) the accessible SOC fraction (2000–20 μm) and (**b**) the mineral-associated SOC fraction (< 20 μm); means and standard errors are shown for *n* = 4 per treatment; soil mass was corrected for differences in bulk density.

